# Between-day reliability of cytokines and adipokines for application in research and practice

**DOI:** 10.3389/fphys.2022.967169

**Published:** 2022-08-22

**Authors:** Grace L. Rose, Morgan J. Farley, Nicole B. Flemming, Tina L. Skinner, Mia A. Schaumberg

**Affiliations:** ^1^ School of Nursing, Midwifery and Social Work, The University of Queensland, Brisbane, QLD, Australia; ^2^ School of Human Movement and Nutrition Sciences, The University of Queensland, Brisbane, QLD, Australia; ^3^ The School of Health and Behavioural Sciences, University of the Sunshine Coast, Sippy Downs, QLD, Australia; ^4^ School of Medicine and Dentistry, Griffith University, Birtinya, QLD, Australia; ^5^ Sunshine Coast Health Institute, Birtinya, QLD, Australia

**Keywords:** inflammation, biological variability, measurement error, biomarkers, agreement

## Abstract

**Purpose:** This study assessed the biological reliability of peripheral human cytokines and adipokines, and the influence of participant characteristics on total error. This has essential application to interventional cytokine measurement to ensure that reported results are interpreted with confidence.

**Methods:** Participants (49% female, 18–85 years, *n* = 84) completed two consecutive-day testing sessions. Participants provided a venous blood sample at the same time of day across two consecutive days, under standardized participant presentation, including 24-h rested and 12-h fasted conditions. Multiplex immunoassay was used to assess inflammatory analytes from samples (predominantly plasma). Repeat measurements were conducted between-day for total precision quantification, and technical (technique) error was negated from the total to provide an estimate of biological (attributed to participant presentation) error.

**Results:** Whilst there was no evidence of statistically significant biological error, a small amount of biological error was consistently present across most analytes (∼3.3%/0.07 pg/ml), which was largest for measurement of leptin (7.3%/210 pg/ml). There was also an influence of sex on reliability of leptin and adiponectin (total model explained 6–7% of error variation), where females demonstrated the greatest error.

**Conclusion:** Biological error reported in this study should be applied to any future study or individual with a repeated measurement of cytokine concentrations over time that maintain best practice procedures (12-h fasted, 24-h rested). In most cases, raw error should be used, with exceptions for women for measurement of leptin and adiponectin. This approach will ensure that results are reported with certainty for improved reporting of intervention efficacy.

## Introduction

Inflammatory cytokines are independently linked to the incidence and progression of numerous common and preventable chronic diseases (St-Onge and Gallagher, 2010; Woods Jeffrey et al., 2012). Given the relationship between chronic inflammation and chronic disease, there is high utility of quantifying cytokines in clinical studies, especially interventional and longitudinal research. Various laboratory techniques are used for the assessment of cytokine concentration (Leng Sean et al., 2008). Regardless of the technique implemented, it is essential that measurement error is minimized for assessment of true inflammatory change in response to longitudinal intervention.

Although technical error (intra-plate coefficient of variation) of biochemistry assays is regularly reported within studies, technical error alone does not consider between-day error of analytes. Given that cytokine and adipokine concentrations are highly influenced by daily fluctuations in diet (Galland, 2010), exercise/physical activity (Pedersen and Toft, 2000), sleep (Irwin Michael and Opp Mark, 2017) and stress (Maydych, 2019), between-day, or biological variability of cytokines is likely substantial, even under standardized conditions.

Beyond longitudinal appraisal (Agalliu et al., 2013; McKay Heather et al., 2017), only two known studies have specifically explored the between-day (Biancotto et al., 2013; Mallard et al., 2020) or diurnal fluctuations (Mallard et al., 2020) of inflammatory cytokines in repeated measures analyses. In these studies, the authors discrepantly report both “high” [co-efficient of variation (CV) = 53.6–73.6%] (Mallard et al., 2020) and “non-significant” between-day cytokine fluctuations (assessed through *t-test*) (Biancotto et al., 2013). However, given the lack of thorough statistical reliability assessment [e.g., intraclass correlation co-efficient (ICC), combined CV] in the study by (Biancotto et al., 2013), and the low sample size (*n* = 10) and homogenous control group reported by (Mallard et al., 2020), it remains unclear what the true influence of day-to-day fluctuations are on cytokine concentrations in a general population. Further, the sole influence of biological error, where technical error is removed from total error to represent purely biological fluctuation, is unclear, and whether participant characteristics (e.g., age and sex) influence biological variability. Biological variability may be greater in older adults and females due to the age-related dysregulation of the immune system that may affect cytokine concentration (Álvarez-Rodríguez et al., 2012) and sex-specific hormone fluctuations that affect cytokine concentration in the peripheral blood and secretion by mononuclear cells (Verthelyi and Klinman, 2000; Ziomkiewicz et al., 2008), respectively.

The present study adds to the body of literature through repeated assessment of cytokine and adipokine concentrations under usual blood collection guidelines (morning, 12-h fasted, 24-h rested), in a large, general population sample of varied age and sex that is more applicable to the general population. This study also provides recommendations for biological error to be applied to longitudinal measurement of cytokines and adipokines within future laboratory analysis.

Therefore, the aims of the present study were to: 1) quantify the biological reliability of peripherally derived cytokines and adipokines within plasma for application to future measurement, 2) assess factors that may influence the between-day reliability of analytes, including participant age and sex. We hypothesized that: 1) biological error would be statistically significant in a positive direction (greater error) when considered separately from technical error, 2) participants of older age and female sex would have greater biological variability, between-days.

## Materials and methods

### Participants

Participant recruitment and study outline have previously been described within publication of the primary study end-point (Rose Grace et al., 2021). Briefly, men and women aged 18–35, 40–60, and 65–85 years volunteered to participate in the study, where a subset were included in the present study (*n* = 84). There were minimal exclusion criteria only relating to exceeding device height and weight restrictions, bodily metal, and water-retention/depletion conditions or medications (Rose Grace et al., 2021). Participants were not excluded based on chronic disease presence. Procedures followed were in accordance with the ethical standards of The University of Queensland and University of the sunshine Coast Human Research Ethics Committees (approvals #2018000547 and #A201362, respectively) and all participants provided written, informed consent.

### Experimental design

Participants underwent two repeated visits within a 26-h period, under identical standardized conditions (overnight fasted ∼12 h, standardized food and fluid intake on day prior, avoidance of moderate to vigorous intensity physical activity for 24 h prior to testing, took normal daily medications (Rose Grace et al., 2021)). Blood collection was completed as the first component of the visit. Body composition measurement was conducted to describe the population, including body mass index (BMI), waist to hip ratio (WHR) and body fat percentage derived from the 4-compartment model (BF%), as previously detailed (Steinfeldt et al., 2013; Rose Grace et al., 2021).

### Plasma and serum collection

Venous blood samples (30–40 ml) were collected from the antecubital vein using a 21-gauge needle into prepared vacutainers by a qualified phlebotomist. A blood draw was taken, once on day 1 and once on day 2, by the same technician and conducted in the morning (6:00a.m.–9:30a.m.) and within 2 hours of the same time of day (26 h). Samples were stored on ice until preparation (30 min), where samples were centrifuged for 10-min at 3,000 rpm following a 20-min coagulation period. Serum and plasma (EDTA) samples were pipetted into individual aliquots and stored at –80°C prior to analysis. Samples underwent nil freeze-thaw cycles prior to analysis.

### Biochemistry analysis

Plasma (*n* = 162) or serum (*n* = 6) samples were analyzed for inflammatory cytokines (IL-1β, IL-6, IL-8, IL-10, IL-12, IL-15, IFN-γ, TNF-α) and adipokines (leptin, adiponectin), using electro chemiluminescent multiplex (Magpix^®^) techniques within the sunshine Coast Health Institute Biochemistry Laboratory. The assays accounted for the low-expected concentration of analytes within general population samples and are accessible within interventional research to determine cytokine concentration [Human High-Sensitivity T-Cell, Human Adipokine (1 and 2), and Human Myokine Magnetic Bead Panels, MILLIPLEX^®^, Merck Millipore, United States ]. Limits of detection were IL-1β: 0.49–2,000 pg/ml, IL-6: 0.18–750 pg/ml, IL-8: 0.31–1,250 pg/ml, IL-10: 1.46–6,000 pg/ml, IL-12 (p70): 0.49–2,000 pg/ml, IL-15: 2–10,000 pg/ml, IFN-γ: 0.61–2,500 pg/ml, TNF-α: 0.45–1,750 pg/ml, adiponectin: 26–400,000 pg/ml, leptin: 38–600,000 pg/ml. Bead vials, serum matrix, quality controls, wash buffer and standards were reconstituted/prepared according to manufacturer instructions within the accompanying protocols (Cat. # HSTCMAG-28SK, HMYOMAG-56K, HADK1MAG-61K, HADK2MAG-61K). Serial dilutions were completed for the preparation of working standards (7 standards), in a 3:1 ratio (150 μL serum matrix diluent: 50 μL stock standard, T-Cell and Myokine panels) or a 4:1 ratio (200 μL serum matrix diluent: 50 μL stock standard, Adipokine panels). Intra- and inter-plate quality controls were included for each plate. Quality controls were compared to the known concentration range, and all fell within the expected concentration.

All samples for each participant were analyzed on the same plate for each assay (96 wells) to eliminate inter-plate variance and were analyzed in duplicate, with the mean taken as the final value for each sample. Final analysis was conducted via Millipore recommended Belysa^®^ software (SigmaAldrich, Darmstadt DEU). Samples were screened against their co-efficient of variation and standard curve-fit and were re-analyzed for concentration as necessary. To account for the two-fold dilution, all samples were multiplied by the dilution factor (x2). For this assessment of reliability, all samples that were below the limit of quantification (BLOQ) (Keizer Ron et al., 2015), had a bead count <35, included a hybrid of serum and plasma mediums (*n* = 3, 55% variance between serum and plasma samples), <75% of data points available for the individual, were extrapolated, or not detected, were omitted. The median technical CV for assays conducted by the technician was 12.1% on average, across all analytes (IL-1β = 11.8%/0.2 pg/ml, IL-6 = 14.8%/0.8 pg/ml, IL-8 = 12.1%/1.0 pg/ml, IL-10 = 12.2%/2.1 pg/ml, IL-12 = 11.0%/0.4 pg/ml, IL-15 = 14.4%/2.5 pg/ml, IFN-γ = 10.8%/1.5 pg/ml, TNF-α = 11.0%/0.6 pg/ml, adiponectin = 24.6%/20807.4 pg/ml, leptin = 12.5%/509.4 pg/ml). These values align with previously published raw technical reliability of Magpix assays, except adiponectin (Biancotto et al., 2013).

### Statistical analysis

Data were analyzed using SPSS^®^ software package (Version 25, IBM Analytics, United States). Age group means and standard deviations for participant characteristics were calculated, a histogram plot was used to determine whether data were normally distributed, a one-way ANOVA/Kruskal Wallis test was conducted to determine age-group differences, and an independent samples *t test*/Mann-Whitney *U* test was used to determine sex differences, depending on normality.

Descriptive statistics of analyte concentration were calculated as medians and quartiles. Total error magnitude (between-day difference) and biological error [between-day minus technical error (difference between duplicate wells of the same sample)] were expressed as percent (relative to concentration) and absolute (raw) values. Absolute confidence intervals (CI; 95%) of raw error and typical error of the estimates (% CV) were also calculated. Systematic bias was assessed as whether 95% CI of the mean difference crossed the line of null effect (value of zero) for both total and biological error and was used to determine whether error was significantly greater than null. Proportional bias of error was evaluated by using a Spearman correlation to compare average measurement values (analyte concentration) against absolute value differences (measurement error), and ICCs were calculated as two-way mixed, absolute agreement.

Total reliability of individual’s raw cytokine/adipokine concentration were assessed via the Bland and Altman method (Bland and Altman, 1986). Bias, upper and lower limits of agreement (LOA) for differences in analyte concentration between-days were calculated. The *a priori* acceptable error value was 15%, in accordance with the threshold used in previous publication that assessed longitudinal intra-individual variation of cytokines (Biancotto et al., 2013). The percentages of participants within acceptable error were reported for each analyte. Techniques were deemed reliable for analysis of individual reliability when 80% of participants met acceptable error limits; chosen to correspond to 95% certainty in technique reliability (0.8 effect size).

The impact of age and sex on magnitude of total error (Log10 transformed) was assessed by hierarchical multiple linear regression to determine the coefficient of determination of each characteristic on measurement error outcome.

## Results

### Participant characteristics

Participant characteristics are presented in Table 1. Participant sex and BMI were consistent across age-groups. Generally, the median pooled inflammatory profile (IL-6, IL-10, TNF-α) was higher compared with a reference apparently healthy, young adult cohort of between 18–39 years (IL-6 = 0.6 pg/ml, *n* = 107; IL-10 = 0.2 pg/ml and TNF-α = 0.6 pg/ml, n = 32) (Ferrucci et al., 2005; Li et al., 2021). Inflammatory cytokine concentrations were similar across all age groups, except IL-15, which was higher among middle-aged adults (*p* = 0.024). Males tended to exhibit elevated levels of many investigated inflammatory cytokines compared with females (IL-1β, IL-6, IL-8, IL-10; *p* = 0.009–0.023), except adiponectin and leptin where females had markedly higher concentrations (*p* < 0.001). As expected, there were age- and sex-related differences in body composition, where young adults had significantly lower BF% and WHR compared to middle-aged and older adults (*p* < 0.001–0.031) and females had higher BF% and WHR compared to males overall (*p* < 0.001). Total caloric intake (*p* = 0.577), fluid intake (*p* = 0.386), and available carbohydrate (*p* = 0.785), total fat (*p* = 0.257) and protein (*p* = 0.393) composition were not different between days 1 and 2.

**TABLE 1:** Participant characteristics.

	Mean/Median	All participants		Young adults		Middle adults		Older adults		Males		Females		p (sex)
		SD/IQR	Mean/Median	SD/IQR	Mean/Median	SD/IQR	Mean/Median	SD/IQR	Mean/Median	SD/IQR	Mean/Median	SD/IQR	p (age)	
n	84	-	27	-	28	-	29	-	43	-	41	-	-	-
Age (years)	49	19.8	25	3.4	52	6.2	72	4.8	50	19.7	50	20.4	-	-
% Female	49	-	48	-	48	-	50	-	0		100		-	-
BMI (kg/m^2^)	25.6	4.3	24.8	3.7	26.4	5.2	25.5	3.7	26.2	3.8	25.0	4.8	0.391	0.207
BF%	29.7	10.0	24.2	9.5	31.0	9.7	33.6	8.8	24.7	8.2	35.3	8.5	**<0.001**	**<0.001**
WHR	0.83	0.09	0.78	0.06	0.85	0.1	0.87	0.09	0.89	0.08	0.78	0.06	**<0.001**	**<0.001**
IL-1β pg/mL^†^	0.7	0.4	0.1	0.3	0.8	0.5	0.7	0.6	0.8	0.6	0.6	0.3	0.337	**0.015**
IL-6 pg/ml^†^	1.7	3.7	1.6	4.8	1.8	4.4	1.7	3.1	2.8	9.2	1.5	1.5	0.956	**0.023**
IL-8 pg/ml^†^	2.6	4.4	2.5	3.8	2.6	4.6	2.7	3.8	3.2	8.0	2.2	1.6	0.771	**0.009**
IL-10 pg/ml^†^	8.3	7.0	7.5	4.7	9.8	8.0	8.6	6.3	9.8	8.4	7.4	5.5	0.352	**0.011**
IL-12 pg/ml^†^	1.8	1.3	1.8	1.4	1.7	1.1	1.8	1.5	1.8	1.8	1.7	1.1	0.650	0.734
IL-15 pg/ml^†^	5.6	3.1	5.6	2.5	6.3	3.4	4.7	2.6	5.5	3.3	5.6	2.3	**0.024**	0.553
IFN-γ pg/mL^†^	7.2	6.2	7.1	5.8	7.6	5.5	6.3	5.2	7.8	6.6	6.4	5.4	0.265	0.163
TNF-α pg/mL^†^	3.2	1.2	3.1	1.1	3.1	0.9	3.6	1.4	3.3	1.3	3.0	1.1	0.120	0.066
APN pg/μL †	30.4	39.4	27.1	36.4	29.7	24.3	42.4	62.2	22.4	20.0	48.3	67.0	0.301	**<0.001**
leptin pg/μL †	1.6	3.4	1.0	2.3	1.9	3.7	1.9	3.6	0.9	1.6	3.0	4.2	0.120	**<0.001**

*n* = 84 unless stated. Descriptive characteristics of pooled, age- and sex-group separated participant characteristics, presented as mean and standard deviation (SD), and Analysis of group characteristic differences by one-way ANOVA (age) and independent samples *t*-test (sex) unless otherwise indicated.

† Presented as median and interquartile range (IQR). Analysis of group characteristic differences by Kruskal-Wallis (age) and Mann-Whitney U (sex) tests.

APN: adiponectin, BF%: body fat percentage, BMI: body mass index, IFN-γ: interferon gamma, IL: interleukin, TNF-α: tumor necrosis factor alpha, WHR: waist to hip ratio.

Bolded values indicate *p* < 0.05.

### Total error of inflammatory analytes

**FIGURE 1:**
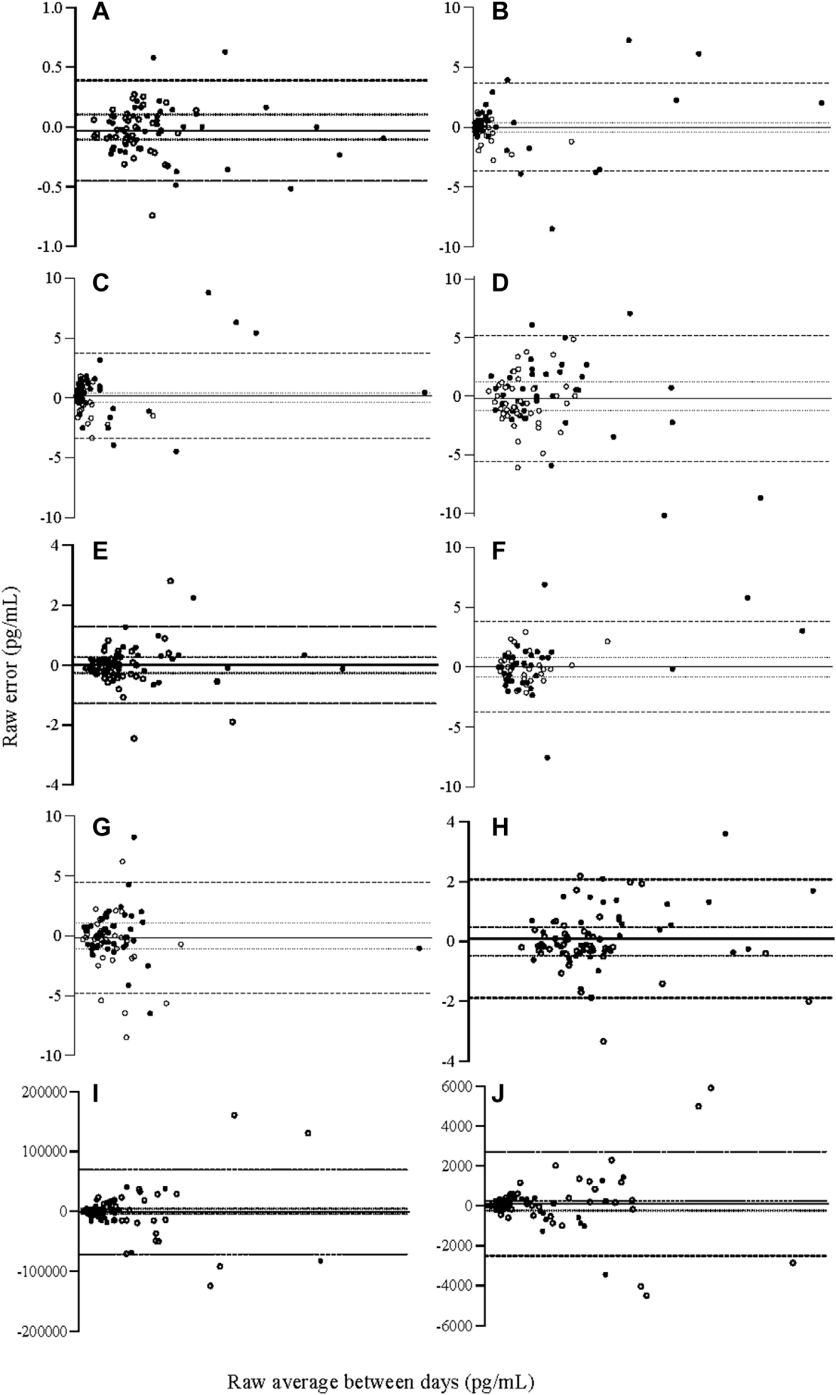
Relative Bland and Altman plots displaying raw between-day (total) error for cytokine analyte concentrations **(A)** IL-1β **(B)** IL-6 **(C)** IL-8 **(D)** IL-10 **(E)** IL-12, **(F)** IL-15, **(G)** IFN-ƴ, **(H)** TNF-α, **(I)** adiponectin, **(J)** leptin. Bias calculated as average error, 95% limits of agreement as two SD from the line of bias, acceptable error of 15% calcualted per analyte, established *a priori*. N = 84. IFN-γ: interferon gamma, IL: interleukin, TNF-α: tumour necrosis factor alpha. ── *Bias, 95% limits of agreement, acceptable error,* ● *males,* ○ *females*.

Systematic bias was not evident when considering the total error of inflammatory analytes (Table 2; 95% confidence intervals of absolute error). There was also no evidence of proportional bias (Table 2; Figure 1). For the measurement of each analyte, 0.0–21.2% of samples were below the limit of quantification, with the highest proportion in measurement of IL-1β (*n* = ∼17). Additionally, 0.0–4.4% of samples were non-detectable, with the greatest proportion for IL-15 (*n* = ∼3; Supplementary Table S1).

**TABLE 2:** Same- and between-day reliability of inflammatory biomarkers, within a general population group (18–85 years, equal sex representation).

	Average values^a^		Average value difference^b^			TEE^c^	Bland and altman^d^		ICC^e^	Proportional Bias^f^	
	Median	Quartiles 25–75%	% of raw	Absolute	95% CI	%CV	% *a priori*	Absolute 95% LOA	r	r	p
**Total error**											
IL-1β (*n* = 79)	0.70	0.56, 0.96	20.89	0.16	-0.08, 0.02	14.77	41.8	-0.45, 0.39	0.953	0.061	0.593
IL-6 (*n* = 83)	1.69	1.16, 4.84	23.96	1.01	-0.40, 0.40	16.95	39.8	-3.64, 3.65	0.994	0.006	0.956
IL-8 (*n* = 84)	2.62	1.82, 6.19	24.08	1.11	-0.21, 0.57	17.03	45.2	-3.39, 3.75	0.993	0.030	0.786
IL-10 (*n* = 84)	8.33	6.30, 13.27	20.10	1.96	-0.80, 0.38	14.21	44.0	-5.61, 5.20	0.955	0.041	0.712
IL-12 (*n* = 84)	1.76	1.31, 2.60	19.11	0.41	-0.13, 0.15	13.51	48.8	-1.28, 1.29	0.968	0.087	0.430
IL-15 (*n* = 65)	5.57	3.92, 7.02	20.42	1.27	-0.45, 0.49	15.03	44.6	-3.77, 3.81	0.856	0.161	0.202
IFN-γ (*n* = 84)	7.20	5.15, 11.38	18.17	1.51	-0.67, 0.33	12.85	52.4	-4.80, 4.46	0.981	0.061	0.583
TNF-α (*n* = 84)	3.16	2.61, 3.76	19.96	0.70	-0.13, 0.30	14.12	52.4	-1.89, 2.07	0.900	0.125	0.256
adiponectin (*n* = 81)	30.39	18.36, 57.80	34.39	19.83	-9.06, 6.65	24.32	27.2	-71.87, 69.46	0.886	0.001	0.990
leptin (*n* = 81)	1.62	0.70, 4.13	24.96	0.70	-0.19, 0.38	17.65	37.0	-2.51, 2.70	0.936	0.111	0.324
**Biological error**											
IL-1β (*n* = 79)	-	-	3.76	0.00	-0.06, 0.06	2.98	-	-	-	-0.142	0.211
IL-6 (*n* = 83)	-	-	3.08	0.17	-0.18, 0.52	2.18	-	-	-	0.049	0.663
IL-8 (*n* = 84)	-	-	6.94	0.16	-0.14, 0.46	4.91	-	-	-	0.045	0.686
IL-10 (*n* = 84)	-	-	2.35	0.00^7^	-0.90, 0.59	1.98	-	-	-	-0.129	0.246
IL-12 (*n* = 84)	-	-	2.49	0.02	-0.10, 0.14	2.32	-	-	-	-0.002	0.988
IL-15 (*n* = 65)	-	-	0.43	0.00^7^	-4.06, 1.47	0.90	-	-	-	-0.068	0.597
IFN-γ (*n* = 84)	-	-	2.88	0.05	-0.52, 0.62	2.04	-	-	-	0.021	0.849
TNF-α (*n* = 84)	-	-	4.48	0.11	-0.05, 0.27	3.17	-	-	-	-0.144	0.191
adiponectin (*n* = 81)	-	-	0.00^7^	0.00^7^	-7.35, 5.40	0.00^g^	-	-	-	0.097	0.391
leptin (*n* = 81)	-	-	7.27	0.21	0.02, 0.40	5.14	-	-	-	0.112	0.321

a Median and 25 and 75% quartiles (IQR) of between-day analyte values for all included participants. Expressed as pg/mL for all analytes except adiponectin and leptin (pg/μL).

b Error calculated as day one minus day two (total/between-day error), and between-day minus same-day (biological error). Error expressed as percentage of the average value from days one and two (% of raw), absolute difference (Absolute) and calculated confidence intervals (95% CI) of absolute difference. Systematic bias was present when intervals did not cross null effect line (0).

c TEE, Typical error of the estimate reported as %CV, coefficient of variation, where the SD, of absolute difference in measures was divided by the absolute mean difference per participant, and averaged.

d Percentage of participants from absolute Bland and Altman analysis that met cut-points of acceptable relative error (*% a priori*) between-days, where a value of 80% was the *a priori* defined lower limit of participants to meet acceptable error for a method to be deemed reliable. *A priori* value was 15%. Absolute 95% LOA, limits of agreement calculated from absolute error data, as two SDs, from the line of bias, where 95% of participants are expected to fall within absolute error limits in future analysis of the investigated inflammatory analytes.

e The absolute agreement of raw error, where a value of >0.8 indicates strong agreement between measurements, completed using two-way mixed ICC, intraclass correlation coefficients.

f The correlative relationship (spearman; r) and significance (*p < 0.05) of the relationship between absolute error and the average absolute value of each analyte.

g Where biological error was calculated to be less than technical error, values were corrected to 0 for average absolute error (Original values IL-10 = -0.15 pg/ml, IL-15 = -1.29, adiponectin = -975.67), percent error (Original values adiponectin = -0.40%) and CV (Original values adiponectin = -0.28%).

IFN-γ: interferon gamma, IL: interleukin, TNF-α: tumor necrosis factor alpha.

For all investigated analytes, none met the 15% cut point that was determined *a priori* (80% of individuals). However, when considered on average, most analytes presented total error that was equal to or less than 15% (IL-1β, IL-10, IL-12, IL-15, IFN-γ, TNF-α; CV = 12.9–15.0%), though some did not (IL-6, IL-8, adiponectin, leptin; CV = 16.96–24.32%). When considered by absolute value rather than percentage, average total error fell between 0.16–1.96 pg/ml, with the exceptions of leptin (699.96 pg/ml) and adiponectin (19831.67 pg/ml) which had greater absolute error than other analytes, but also higher absolute concentration (Table 2).

### Biological error of inflammatory analytes

Biological error was greater than technical error of the assay in most cases, where the total CV was increased by 0.9–5.1% across all analytes except adiponectin (no difference in CV; Table 2, Biological error). However, there was no evidence of systematic bias in a positive direction to indicate that biological variances in analytes had a statistically significant influence on measurement error beyond technical error of the measurement (95% CIs of raw biological error, Table 2). Although biological error contributed to an increase in the total CV, increases in raw biological error were between 0.00–0.17 pg/ml, except for leptin (210 pg/ml Table 2). There was also no evidence of proportional bias (Table 2).

### The influence of age and sex on total error

Multiple regression analysis indicated that age and sex had minimal influence on total measurement error, where technical and biological were not separated (Table 3). There was some influence of sex and age on cytokine measurement error, where those who demonstrated greater error for IL-6 were male, and for IFN-γ were of younger age. However, the influence of age and sex on total cytokine measurement error was small, where these significant associations explained only 6–7% of measurement error variation among the cohort. Generally, for measurement of cytokines, the overall model did not significantly explain daily measurement variance [range 1–7% of variance (average 4%)].

**TABLE 3:** Impact of age and sex on raw total error of cytokines and adipokines^1^.

Outcome	Total *r* ^2^	Associations	β ^b^	95% CI	p ^c^
IL-1β	0.01	Constant	-0.92	-1.19, -0.66	**<0.001**
		Sex	0.02	-0.17, 0.02	0.858
		Age	-0.02	-0.14, 0.10	0.735
IL-6	0.06	Constant	-0.08	-0.46, 0.30	0.674
		Sex	-0.28	-0.55, -0.01	**0.045**
		Age	-0.08	-0.25, 0.09	0.355
IL-8	0.04	Constant	-0.08	-0.41, 0.25	0.631
		Sex	-0.20	-0.43, 0.03	0.091
		Age	-0.03	-0.18, 0.11	0.666
IL-10	0.05	Constant	0.42	0.09, 0.74	**0.013**
		Sex	-0.14	-0.37, 0.10	0.242
		Age	-0.12	0.03, -0.19	0.106
IL-12	0.02	Constant	-0.42	-0.74, -0.11	**0.009**
		Sex	0.01	-0.21, 0.22	0.933
		Age	-0.09	-0.23, 0.04	0.171
IL-15	0.07	Constant	0.24	-0.12, 0.61	0.191
		Sex	-0.15	-0.40, 0.10	0.223
		Age	-0.13	-0.28, 0.04	0.122
IFN-γ	0.07	Constant	0.30	-0.02, 0.62	0.066
		Sex	-0.10	-0.33, 0.12	0.369
		Age	-0.16	-0.31, -0.02	**0.024**
TNF-α	0.03	Constant	-0.19	-0.46, 0.08	0.159
		Sex	-0.12	-0.31, 0.07	0.214
		Age	-0.06	-0.17, 0.06	0.354
adiponectin	0.11	Constant	3.59	3.20, 3.97	**<0.001**
		Sex	0.40	0.13, 0.67	**0.004**
		Age	0.06	-0.11, 0.23	0.455
leptin	0.18	Constant	1.83	1.42, 2.25	**<0.001**
		Sex	0.58	0.29, 0.87	**<0.001**
		Age	0.13	-0.05, 0.32	0.149

a Influence of age and sex on total absolute error of analytes (all Log10 transformed, IL-15 *n* = 61; IL-1β n = 76, IL-10 *n* = 80; IL-6, adiponectin *n* = 81; IL-12 *n* = 82; IL-8, IFN-γ *n* = 83; TNF-α, leptin *n* = 84) analyzed within combined multiple regression models, where the influence of the overall model is presented as the coefficient of determination (*r* ^2^).

b Unstandardized beta (β) reported.

c Significance (*p* < 0.05) of the model components’ influence (age and sex) on absolute total error, 95% CI, confidence intervals of absolute (Log10 transformed) error reported.

IFN-γ: interferon gamma, IL: interleukin, TNF-α: tumor necrosis factor alpha.

Bolded values indicate *p* < 0.05.

For measurement of adipokines, there was an influence of sex on total measurement error, where females demonstrated greater error than males during between-day measurement of adiponectin and leptin (Table 3). The influence of the overall model, including sex, explained 11–18% of variation for adiponectin and leptin respectively. The influence of sex is also demonstrated in Figures 1I, J, where the largest measurement error for adiponectin and leptin was seen among women.

## Discussion

The purpose of this study was to quantify biological error of commonly measured inflammatory biomarkers for application in clinical trial and longitudinal inflammatory outcomes. Overall, there was no evidence for significant biological error (between-day variability attributed to participant presentation) when technical error of the measurement was removed from total error of the measurement. However, small changes in biological error must still be considered for best practice assessment of reliability. On average, measurement error increased by 3.3% (maximum 6.9%) and 0.07 pg/ml (maximum 0.17 pg/ml) for common inflammatory cytokines when error was considered between days. The exception to this was leptin, where an addition of 7.3%/210 pg/ml was seen. Consideration of biological error in addition to laboratory-specific technical measurement error will help establish whether change reported in trials exceeds the total error of the measurement and enhance the veracity of reported outcomes for both individual (responder and non-responder) and group-level comparison analyses, including group differences in controlled trials.

Although cytokine concentration appeared to be higher in this population compared with referent young and healthy populations (Ferrucci et al., 2005; Li et al., 2021), the absolute concentration of most analytes was relatively low. When compared with apparently healthy populations of mixed age-groups, concentrations of IL-6 (6.5 pg/ml, median age = 37 years, n = 228) (Fernandez-Real et al., 2001) and IL-10 (12.6 pg/ml, median age = 36 years, n = 35) (Kleiner et al., 2013) were lower than expected. This is especially important given that, whilst percent error and CV appeared high in this sample and total error exceeded the criteria for the *a priori* minimum of 15%, absolute biological error of the measurement was less than 0.1 pg/ml on average for most analytes.

Given the low inflammatory status of the cohort, absolute error should be considered more highly than percentage error, in practice. Considering there was no evidence of proportional bias, absolute error can be applied across the span of lower to higher concentrations of inflammatory analytes with confidence. Percent error is commonly reported and applied and may be necessary for reporting change among individuals. However, this approach may lead to inflation of or underreporting of error values when applied to populations or individuals with higher or lower inflammatory status, respectively. This is because if percent biological error reported here (∼3.3%) was applied to a population with heightened cytokine concentration, it would result in much greater raw error than in a population with lower cytokine concentration. The only instance where it may be more appropriate to use percent error is among populations with very low cytokine concentration and greater assay sensitivity, to ensure that total error falls within the measured concentration.

Only two known studies have specifically assessed between-day cytokine variability. Although it is not possible to compare the CVs from Biancotto et al. (Biancotto et al., 2013), who considered days of testing separately, Mallard et al. (Mallard et al., 2020) reported CVs of healthy individuals (n = 10) that were far greater than reported among the present data (CV: IL-6 = 59.9%, IL-8 = 53.6%, IL-10 = 63.7%, TNF-α = 73.6%). However, likely due to the inclusion of participants with lower concentrations of inflammatory analytes than the current study, error relative to the measured value (percent error) was inflated, by 3-fold and 4.5-fold for IL-10 and IL-6, respectively (Mallard et al., 2020). Additionally, it was not reported whether participants were fasted for sample collection, which is known to influence the concentration of circulating inflammatory cytokines (Galland, 2010). These discrepancies, along with the smaller sample size, may explain why total CV reported in the current study is lower than previously reported.

In chronic disease populations (e.g., type-two diabetes), reported measurement error is not significantly different to comparator healthy populations (Mallard et al., 2020). Therefore, it is likely that the results of the present study may also be applied to clinical populations. However, there are some considerations, including the greater possibility of acute illness across an intervention and variability in health status (e.g., glycemic control in diabetics (Collier et al., 2008), treatment type and duration in oncology populations (Schauer et al., 2021)). Therefore, whilst considering whether interventional change of future trials exceeds the total error of the present study may assist in determining whether change is real, it is important to do so with the awareness that error may be greater among those with different clinical presentation from beginning to end of an intervention. Further research is required to determine how much additional variability should be expected among clinical populations for more specific application.

When considering the influence of participant characteristics on total error, there was a significant influence of sex for both leptin and adiponectin, where women tended to demonstrate the greatest error. Whilst age and sex were significant in multiple linear regression for other cytokines, they only explained a maximum of 6% of the sample variability. Interestingly, in addition to greater error, women had higher average concentrations of both adipokines when compared with men (Table 1). When visually assessing Bland and Altman plots of leptin and adiponectin (Figures 1I, J), it appeared that the influence of sex could mainly be explained by women with a higher basal concentration of both adipokines. Therefore, although there was no evidence of proportional bias when assessed statistically, it is possible that error seen among women became more pronounced in magnitude when basal concentrations of analytes were higher. This could be due to the influence of sex hormones on adipokines (Karim et al., 2015), where women with a higher body fat percentage often have higher adipokine concentrations (Smith et al., 2006) as well as circulating estrogen (Ziomkiewicz et al., 2008). It is possible that daily fluctuations of sex hormones (Panico et al., 1990), especially among women with higher concentrations of adipokines, could magnify the variability seen, though this cannot be confirmed from the present analysis. Therefore, it is recommended that percent variability reported in this study be applied for measurement of adiponectin and leptin in women to account for the possible greater variability seen among women with higher adipokine concentrations to ensure a greater degree of certainty in outcome reporting.

In assessing biological and total error of analytes, is it essential to concurrently consider the application of clinically meaningful limits so this value can be added to estimates of error. At present, there are very few established clinically meaningful limits for inflammatory cytokines. For changes in chronic inflammation, IL-6 is the only analyte with a minimally clinically important difference (MCID) bench mark of 1.70 pg/ml (Andrews James et al., 2017). To our knowledge, whilst there are no established MCIDs for other investigated analytes, results from small sample linear regression analysis indicate that changes in IL-1β by 0.54 pg/ml may be clinically meaningful for reductions in cancer incidence in a clinical population (chronic pulmonary disease) (Zhan et al., 2018). Overall, analytes met the current clinically meaningful limits, where limits of agreement of this analysis were below limits for IL-1β, and IL-6 between-day error was below the meaningful threshold on average. This is promising; however, it is important to consider that clinically meaningful limits are likely population dependent, especially considering age and chronic disease status. It is essential that future research investigates suitable limits for future application.

Importantly, clinically relevant variability values for biochemistry, particularly cytokine analysis, are seldom considered when reporting the magnitude of change in response to an intervention (e.g., diet, pharmacological, sleep, exercise). For example, in a recent systematic review conducted by our lab (Rose Grace et al., 2020), only 41% of studies reported intra-assay CV of cytokines, and none considered clinically meaningful change. Of the few studies that found a significant influence of exercise intensity on cytokine concentrations (Abd El-Kader et al., 2013; Santos Fabio et al., 2017; Gerosa-Neto et al., 2019), no study applied CV or meaningful change to their results. Whilst one study found a large interventional effect that was well beyond error reported here (Abd El-Kader et al., 2013), one study was very close to falling below error limits (IL-6 = 28.8%/1.10 pg/ml change) (Gerosa-Neto et al., 2019), and the significant change reported in another study fell below error reported here (IL-10 = 1.21 pg/ml change) (Santos Fabio et al., 2017). This further confirms the necessity of considering reliability of the measurement technique and between-day participant variation, along with clinically meaningful limits for confidence in reporting of results.

There are several limitations of the present study that must be considered. First, although technical error was in the range of what was expected when considering absolute error (particularly important due to the low concentration of analytes), it was greater than the reported attainable CV from the manufacturer (Millipore). This may mean that true biological error of the assay was clouded due to inflated technical error. However, technical error of the assay was comparable to results previously seen in a similar multiplexed analyses that aimed to assess biological variability (Biancotto et al., 2013). Additionally, analyses within this study were completed by hand as opposed to robot pipetting. However, due to the capability of many laboratories, automated analyses are not commonly routine, therefore this study is of relevance to a large body of research, including previously published studies. We also considered variability over 1 day only. This design was necessary to assess acutely influential factors (e.g., food, physical activity) on total cytokine error, but it is possible that with more days between assessments, error may be greater due to the multitude of chronic variables that may change over the course of an intervention (e.g., illness (Arango and Descoteaux, 2014), medication change (Lisboa Felipe et al., 2017). The possible influence of other chronic biological changes should be considered, and where possible measured, in addition to the total error reported here.

Overall, the application of biological error reported in this study should be considered within future reporting of repeated cytokine measurement over time, in addition to the technical error of the investigating laboratory, to derive total error. Total error described for each analyte in this study can also be used as an estimate of between-day reliability and applied to longitudinal appraisal. Specifically, if total error exceeds repeated measurement change of an intervention, we suggest that results should be interpreted cautiously, given the day-to-day variation noted in this study, even when many influential variables were controlled.

When considering the error value (absolute or relative) to include within longitudinal evaluation, inflammatory status and sex may be important factors. The present findings indicate that absolute raw error is likely most appropriate in application of biological error in most cases. However, for participants who have very low cytokine concentrations (below referent young population) or women for measurement of leptin and adiponectin, the use of percentage may be more appropriate. It is also essential that pre-testing guidance, including resting and fasting preparation, be adhered to wherever possible. This undoubtably reduces biological variability, likely similar to levels reported within this study, as nutrition and exercise are key modulators of inflammatory cytokine concentrations (Pedersen and Toft, 2000; Galland, 2010). In instances where pre-testing controls cannot be implemented, it is important to be aware that biological error may be greater than what is reported in this study. In conclusion, the implementation of these recommendations are essential to accurate reporting of inflammatory outcomes for interventions and individuals and should be applied wherever possible.

## Data Availability

The raw data supporting the conclusion of this article will be made available by the authors, without undue reservation.
