# Supplementary material for: Between-day reliability of cytokines and adipokines for application in research and practice
Source: Front Physiol. 2022 Aug 22;13:967169. doi: 10.3389/fphys.2022.967169 (PMC9444151; doi:10.3389/fphys.2022.967169)
Supplement: Supplementary file 1 [file Table1.DOCX]

**Supplementary Table 1.** Number and percentage of samples below limit of quantification (BLOQ)

|  | Replicates^1^  (n=) | BLOQ^2^  (n=) | BLOQ^2^  (%) | ND^3^  (n=) | ND^3^  (%) |
| --- | --- | --- | --- | --- | --- |
| **IL-1β (n=79)** | **312** | **66** | **21.15** | **0** | **0.00** |
| *Day 1 (1, 2)* | *78, 79* | *16, 19* |  | *0, 0* |  |
| *Day 2 (1, 2)* | *76, 79* | *17, 14* |  | *0, 0* |  |
| **IL-6 (n=83)** | **328** | **1** | **0.31** | **2** | **0.61** |
| *Day 1 (1, 2)* | *83, 83* | *0, 0* |  | *0, 0* |  |
| *Day 2 (1, 2)* | *81, 81* | *1, 0* |  | *1, 1* |  |
| **IL-8 (n=84)** | **335** | **0** | **0.00** | **0** | **0.00** |
| *Day 1 (1, 2)* | *84, 84* | *0, 0* |  | *0, 0* |  |
| *Day 2 (1, 2)* | *84, 83* | *0, 0* |  | *0, 0* |  |
| **IL-10 (n=84)** | **334** | **0** | **0.00** | **0** | **0.00** |
| *Day 1 (1, 2)* | *83, 83* | *0, 0* |  | *0, 0* |  |
| *Day 2 (1, 2)* | *84, 84* | *0, 0* |  | *0, 0* |  |
| **IL-12 (n=84)** | **335** | **1** | **0.30** | **0** | **0.00** |
| *Day 1 (1, 2)* | *84, 83* | *0, 1* |  | *0, 0* |  |
| *Day 2 (1, 2)* | *84, 84* | *0, 0* |  | *0, 0* |  |
| **IL-15 (n=65)** | **248** | **9** | **3.63** | **11** | **4.44** |
| *Day 1 (1, 2)* | *61, 61* | *3, 3* |  | *4, 2* |  |
| *Day 2 (1, 2)* | *67, 59* | *0, 3* |  | *0, 5* |  |
| **IFN-γ (n=84)** | **336** | **0** | **0.00** | **0** | **0.00** |
| *Day 1 (1, 2)* | *84, 84* | *0, 0* |  | *0, 0* |  |
| *Day 2 (1, 2)* | *84, 84* | *0, 0* |  | *0, 0* |  |
| **TNF-α (n=84)** | **336** | **0** | **0.00** | **0** | **0.00** |
| *Day 1 (1, 2)* | *84, 84* | *0, 0* |  | *0, 0* |  |
| *Day 2 (1, 2)* | *84, 84* | *0, 0* |  | *0, 0* |  |
| **adiponectin (n=81)** | **313** | **1** | **0.32** | **0** | **0.00** |
| *Day 1 (1, 2)* | *78, 80* | *0, 0* |  | *0, 0* |  |
| *Day 2 (1, 2)* | *78, 77* | *1, 0* |  | *0, 0* |  |
| **leptin (n=81)** | **316** | **0** | **0.00** | **0** | **0.00** |
| *Day 1 (1, 2)* | *78, 79* | *0, 0* |  | *0, 0* |  |
| *Day 2 (1, 2)* | *80, 79* | *0, 0* |  | *0, 0* |  |

^1^ Total number of participants with analytes that were measured for two replicates (bloods analyses completed in duplicate), from day one and day two of blood sampling.

^2^ Of the total number of participants with measured replicates, the number (n=) and proportion (%) that fell below the limit of quantification (BLOQ) for each analyte, in total and calculated separately for sample day one and two

^3^ Of the total number of participants with measured replicates, th1e number (n=) and proportion (%) that were non-detectable (ND) for each analyte, in total and calculated separately for sample day one and two

IFN-γ: interferon gamma, IL: interleukin, TNF-α: tumor necrosis factor alpha
